# Editorial: Defeating HIV in Africa: an achievable goal

**DOI:** 10.3389/fpubh.2026.1904941

**Published:** 2026-08-07

**Authors:** Stefano Orlando, Kristien Wouters, Massimo Leone, Maria Cristina Marazzi

**Affiliations:** 1 Department of Biomedicine and Prevention, Università degli Studi di Roma Tor Vergata, Rome, Italy; 2 Instituut voor Tropische Geneeskunde, Antwerp, Belgium; 3 Fondazione IRCCS Istituto Neurologico Carlo Besta, Milan, Italy; 4 Disease Relief through Excellent and Advanced Means (DREAM) Program, Community of S. Egidio, Rome, Italy

**Keywords:** antiretroviral therapy (ART), differentiated service delivery, drug resistance, HIV/AIDS, sub-Saharan Africa

## 1 Introduction

Over the last four decades, the global response to HIV/AIDS has represented one of the most significant achievements in modern public health. A disease once perceived as inevitably fatal has been transformed, for millions of people, into a chronic and manageable condition. This transformation was not the result of biomedical innovation alone. It was made possible by an unprecedented convergence of scientific progress, political commitment, international financing, community engagement, social mobilization, and the active involvement of civil society and affected communities (1). The creation of dedicated global institutions, the mobilization of large-scale funding mechanisms, the reduction in the price of antiretroviral medicines, and the expansion of treatment and prevention services have made the AIDS response a paradigmatic example of how the international community can confront a shared health threat (2, 3).

Yet this success remains unfinished and fragile. In 2024, an estimated 40.8 million people were living with HIV worldwide, 1.3 million people acquired HIV infection, and 630,000 people died from AIDS-related illnesses (4). Despite major reductions in AIDS-related mortality and new infections since 2010, the world remains far from the goal of ending AIDS as a public health threat by 2030 (5, 6). The current moment is particularly critical because the epidemiological challenge is now compounded by a political and financial one. Donor fatigue, shifting geopolitical priorities, constrained national budgets, and a broader crisis of multilateral cooperation are threatening the continuity of programmes that depend on sustained long-term investment (7). Reductions or disruptions in international financing risk undermining prevention, treatment, laboratory monitoring, community-led services, and health system capacities built over decades (8, 9).

The risk, however, is not only a reduction in available resources. It is also the possibility that a complex, long-term, and deeply social health challenge may be addressed through overly simplified responses. The history of HIV/AIDS shows that there are no simple solutions to complex public health problems. The expansion of antiretroviral therapy required not only drugs, but laboratories, supply chains, trained health workers, adherence support, community trust, human rights-based approaches, and sustained clinical follow-up (10). Prevention required not only technologies, but education, stigma reduction, gender-sensitive interventions, and access to services for marginalized populations (11, 12). The current phase of the epidemic requires the same level of complexity in thinking and action.

This is particularly evident in two areas that are central to the future of HIV care: antiretroviral drug resistance and differentiated service delivery. The emergence of resistance to dolutegravir, a cornerstone of current first-line treatment, is not only a clinical warning. It also raises broader questions about how health systems monitor, accompany, and support people living with HIV over the long term. Similarly, differentiated service delivery is essential for making services more responsive, accessible, and sustainable, but it should not be reduced to fewer contacts, weaker clinical oversight, or lower standards of care. Differentiation should mean tailoring services to people's needs, preserving quality and continuity, and identifying those who require more intensive support. It should not become a minimalist model of care for people living in poorer settings (13).

For this reason, the current debate on HIV/AIDS is also a debate on the future of global health. The AIDS response has helped shape many of the principles now considered essential for effective health systems: equity, universal access, community participation, integration of services, chronic care, laboratory capacity, attention to social determinants, and protection of vulnerable populations. At the same time, the challenges now faced by HIV programmes—declining political commitment, fragmented governance, financial uncertainty, drug resistance, comorbidities, and persistent inequalities—mirror the broader vulnerabilities of global health cooperation. The way the international community responds to this moment will therefore have implications that extend beyond HIV.

The *Defeating HIV in Africa: an achievable goal* conference, organized in Rome in January 2025 by the Community of Sant'Egidio (14), and this Research Topic are timely and relevant precisely because they address this crossroads. Starting from the medical and scientific realities of HIV care, the historical experience of the global AIDS response, and concrete programme experience in resource-limited settings, they invite a wider reflection on the future of health cooperation. Ending AIDS requires renewed investment, but also renewed vision. It requires innovation, but also continuity. It requires efficiency, but not simplification at the expense of quality. Above all, it requires the recognition that sustainable progress in global health depends on the capacity to combine science, solidarity, resilient health systems, and person-centred care.

## 2 The articles in the Research Topic

The article by Orlando et al. provides the foundational socio-economic and historical framework for this Research Topic. While unprecedented global investments successfully transformed HIV into a manageable chronic condition, the resulting increase in patients requiring lifelong antiretroviral therapy now severely strains resource-constrained health systems. In high-burden countries, the financial requirements to sustain universal HIV care frequently exceed total national public health expenditures.

Compounded by a documented decline in international funding since 2017, this macroeconomic vulnerability frames the core clinical challenges addressed in the subsequent articles. The authors warn that financial pressures are driving a dangerous shift toward excessively simplified care models. While intended to expand access, these paradigms risk compromising virological monitoring and fostering drug resistance. Ultimately, Orlando et al. demonstrate that sustaining high-quality HIV care is not an isolated financial burden but a critical multiplier for broader health system resilience, warning against the long-term systemic costs of short-term financial retrenchment.

Providing an authoritative institutional perspective, Sands, Executive Director of the Global Fund, evaluates the critical juncture of the global HIV response. Drawing on the historic successes of multilateral mobilization—which dramatically expanded treatment access and saved millions of lives—Sands warns against the current erosion of political will and international funding. He argues that premature financial withdrawal represents a dangerous “false economy” that threatens to dismantle decades of hard-won progress, particularly as HIV incidence rises in new geographic regions and structural barriers persist. Highlighting the pivotal 2025 Global Fund Replenishment, Sands emphasizes that navigating the “last mile” of the epidemic demands renewed global solidarity, rapid access to biomedical innovations, and a carefully managed, gradual transition toward resilient, domestically financed health systems.

The contributions by Ciccacci et al. and Perno et al. articulate the foremost clinical and systemic challenges currently threatening the global HIV response, centering on the alarming emergence of dolutegravir (DTG) resistance. Both studies argue that this resistance is not an isolated clinical anomaly, but a profound systemic warning signal driven by the oversimplification of care models in resource-constrained settings. Perno et al. emphasize that the programmatic goal of HIV care must fundamentally shift from merely achieving to durably maintaining lifelong virological control. They warn that inadequate monitoring and the recycling of compromised drug backbones inevitably lead to functional monotherapy and resistance, stressing that diagnostics—such as viral load and genotypic resistance testing—must be treated as essential healthcare infrastructure rather than optional add-ons. Complementing this virological perspective, Ciccacci et al. caution against the recent push toward extreme decentralization and “one-size-fits-all” service delivery, which risks depersonalizing care and eroding critical clinical oversight. Together, these articles call for a paradigm shift away from minimalistic, cost-driven approaches. They advocate instead for high-quality, differentiated service delivery, expanded diagnostic capacity, and robust community support to ensure resilient, person-centered lifelong care.

Concluding the Research Topic, the article by Doro Altan et al. provides a concrete, field-tested paradigm by detailing the 20-year experience of the DREAM Programme run by Community of Sant'Egidio. Directly intercepting the systemic and clinical challenges highlighted in the previous articles, this contribution offers a practical model for implementing high-quality, person-centered HIV care in resource-constrained settings. By explicitly rejecting diagnostic and therapeutic minimalism, the program demonstrates that robust virological monitoring, sustained patient retention, and the integration of broad chronic disease management are practically achievable. The DREAM model illustrates that combating emerging threats, such as dolutegravir resistance, requires moving beyond oversimplified, “one-size-fits-all” protocols. Instead, it demands sustained investment in comprehensive diagnostic infrastructure, community empowerment, and trusting patient-provider relationships. Ultimately, this article serves as a powerful operational blueprint, proving that equitable, high-standard lifelong care is not only feasible in sub-Saharan Africa but remains the only sustainable path forward for the future of global health.

## 3 Conclusions and future directions

Ultimately, the insights presented in this Research Topic compel a paradigm shift in how global and national public health programs conceptualize the final phase of the HIV/AIDS response. At the international level, navigating the “last mile” of the epidemic demands a transition from emergency-driven aid to sustainable, predictable financing, avoiding the catastrophic consequences of premature donor withdrawal. Nationally, health systems must pivot from minimalistic, cost-driven delivery models toward high-quality, differentiated care frameworks that prioritize lifelong virological control rather than mere initial suppression. This necessitates embedding advanced diagnostics—specifically routine viral load monitoring and genotypic resistance testing—as essential, non-negotiable clinical infrastructure rather than optional surveillance tools. Furthermore, national programs must leverage existing HIV investments as multipliers for broader health system resilience by actively integrating HIV care with the management of emerging non-communicable diseases and chronic comorbidities.

Looking forward, these contributions outline vital trajectories for future research. Investigations must prioritize the longitudinal evaluation of differentiated service delivery models to assess their true impact on clinical outcomes, the emergence of drug resistance, and patient retention, particularly among vulnerable and marginalized populations. Additionally, operational and health economic research is urgently needed to identify scalable, cost-effective strategies for deploying genotypic resistance testing and managing multi-class treatment failure in resource-constrained settings. Finally, as long-acting antiretroviral therapies (LA-ART) enter the global landscape, implementation science must establish rigorous, context-specific frameworks to safely integrate these biomedical innovations without compromising their genetic barrier or inadvertently fueling further drug resistance.

Ending AIDS as a public health threat remains an achievable goal, but only if the global community commits to preserving therapeutic efficacy through equity, resilient health infrastructures, and deeply person-centered innovation.
